# Abdominal wall incisional hernia repair improves respiratory function: results after 3 years of follow-up

**DOI:** 10.1007/s10029-020-02302-7

**Published:** 2020-09-14

**Authors:** L. Licari, S. Campanella, C. Carolla, C. Madonia, B. Canino, G. Salamone

**Affiliations:** 1 Department of Surgical, Oncological and Oral Sciences, Policlinico P. Giaccone, University of Palermo, Via Liborio Giuffré 5, 90127 Palermo, Italy; 2 Department of Health Promotion, Mother and Child Care, Internal Medicine and Medical Specialties, Policlinico P. Giaccone, University of Palermo, 90127 Palermo, Italy

**Keywords:** Incisional hernia, Surgery, Ventral hernia repair, Respiratory outcome

## Abstract

**Purpose:**

Hernias severely impact patient quality of life (QoL), and 80% of patients require a surgical operation. Moreover, hernias are responsible for respiratory function alterations. This study aims to investigate the postoperative alterations in respiratory function after open ventral hernia repair in patients with incisional hernia.

**Methods:**

Patients operated on at the Policlinico “Paolo Giaccone” at Palermo University Hospital between January 2015 and December 2016 were identified in a prospective database. Fifty-one patients were enrolled in the study. The respiratory outcome measures used were forced vital capacity (FVC), forced expiratory volume in 1 s (FEV1), FEV1/FVC ratio, peak expiratory flow (PEF) and PEF percentage (%PEF). The timepoints at which the parameters listed were assessed were *t*_0_, 1 week before the surgical operation; *t*_1_, 12 months later; and *t*_2_, 3 years later.

**Results:**

The difference between mean preoperative and postoperative PEF was significant [*t*_0_ 4.32 (4.03–7.92), *t*_1_ 6.7 (4.27–8.24) with *p* = 0.012 and *t*_2_ 6.5 (4.25–8.21) with *p* = 0.026]. The %PEF increased from 75% preoperatively to 87% at *t*_1_ (*p* = 0.009) and to 85% at *t*_2_ (*p* = 0.03). No differences were found in the comparison of pre- and postoperative FVC, FEV1 or FEV1/FVC ratio.

**Conclusion:**

The improvement in respiratory measures suggests the importance of abdominal wall restoration to recover functional activity of respiratory function.

## Introduction

The estimated incidence of incisional ventral hernia is approximately 20%, increasing to 40% in high risk populations [1].

The indications for surgical operation are pain, functional limitations and poor appearance due to bulging. Surgery should provide not only anatomical restoration of the abdominal wall, but should also improve pulmonary and postural functions. The magnitude of this field of interest is significant, as 80% of patients require surgical operation [2].

Incisional ventral hernia is responsible for considerable anatomical and physiological alterations in the skeletal muscle system, resulting in alterations in quality of life (QoL). The presence of incisional ventral hernia could be responsible for significant alterations in respiratory mechanism functions [3].

The real impact of incisional ventral hernia and consequently of abdominal wall restoration on respiratory mechanisms is currently unknown because of the absence of homogeneous clinical data focused on this topic.

This study aims to investigate the postoperative alterations in respiratory function after open ventral hernia repair.

## Patients and methods

Patients with abdominal wall incisional hernia treated with surgical operation at the Policlinico “Paolo Giaccone” at Palermo University Hospital between January 2015 and December 2016 were identified in a prospective database and the data collected were retrospectively reviewed. Approval by the Regional Ethics Review Board was obtained (ID number 0–201-9–05). Patients’ medical and surgical records were collected from the charts and surgical registries. The diagnosis of abdominal wall incisional hernia was obtained after physical examination and US/CT scans. The inclusion criteria were a primary incisional hernia requiring elective surgical treatment, age between 18 and 85 y.o., abdominal wall defect ≥ 5 cm and patient willingness to participate in the study. No patients underwent other surgical operations during the follow-up time. Urgent surgical operations, recurrent incisional hernias, pre-existing pulmonary comorbidities and musculoskeletal disorders affecting daily activities were exclusion criteria. The surgical operation performed was the open intraperitoneal onlay mesh (IPOM) positioning technique, which represents a highly standardised technique for complex abdominal wall hernias.

All operations were performed by one skilled general surgeon with patients under general anesthesia receiving preoperative antibiotic prophylaxis (ceftazidime 2 gr at least 1 h before the skin incision). For all surgical operations, a three-dimensional (3D) textile monofilament polyester (PET) mesh with bioabsorbable collagen film (pore size 3.3 mm 92.3 mm, density 66 g/m^2^, thickness 0.7 mm) was used (Symbotex, Covidien). The midline was always reconstructed with complete covering of the prosthesis. No bridging mesh or component separation technique was used. The dimensions of the mesh used were large enough to cover the fascia 5 cm over the hernia defect borders (overlap) in all directions. The European Hernia Society (EHS) classification was obtained taking into account the site of the hernia, the width of the defect and the number of orifices. A follow-up was performed for at least 3 years. After discharge from the hospital, all patients were examined weekly during the first month, monthly for 6 months thereafter and then annually. The demographic preoperative data collected were age; sex; body mass index (BMI); tobacco use; comorbidities including diabetes, hypertension, cardiovascular diseases, pulmonary diseases and liver diseases; and risk group according to the American Society of Anesthesiologists (ASA).

The intraoperative data evaluated were the duration of the operation (skin-to-skin time) and the hernia size. Regarding the postoperative data, in-hospital stay (IHS) and complications were evaluated. The complications were divided into two groups: early complications (up to 30 postoperative days), such as wound infection, haematoma and seroma, and late complications, including hernia recurrence.

The spirometric measures—forced vital capacity (FVC), forced expiratory volume in 1 s (FEV_1_), FEV_1_/FVC ratio, peak expiratory flow (PEF) and PEF percentage (%PEF)—were used to evaluate the respiratory outcome.

The timepoints at which the parameters listed above were assessed for each patient involved in the study were 1 week before the surgical operation (*t*_0_) and 12 months (*t*_1_) and 3 years (*t*_2_) later.

Spirometry was performed via the measurement of lung function with the patient in a standing position using the Sensomedics V_max_^®^ Encore spirometer. Spirometry was performed once per patient at each time point.

The data were analysed in Excel 2016 and IBM SPSS software, version 21. The data distribution was tested for normality using the Shapiro–Wilk test. The mean and median (range) were obtained for continuous variables. Comparisons of continuous variables were performed using paired Student’s *t* test. A comparison of categorical variables was performed with the Chi squared (*χ*^2^) test or Fisher’s exact test. The statistical significance level was set to a *p* value < 0.05. Comparison between the preoperative mean of each spirometric measure at *t*_0_ and the corresponding postoperative means at *t*_1_ and *t*_2_ was performed. A short description of the spirometric functional outcome measures is listed below.

### Surgical technique

The surgical operation was executed under general anaesthesia. Antibiotic prophylaxis was administered at least one hour prior to beginning the operation. The WHO surgical safety checklist was used to verify the correct patient, procedure and site and additional critical information prior to beginning the procedure. The anterior abdominal wall was disinfected with chlorhexidine for surgical skin preparation. A vertical midline incision incorporating the old incision was made. The old scar was completely excised. The incision proceeded to the fascia. The hernia sac was identified and dissected from the surrounding tissue. The sac was opened. The adhesions between the gut and the sac were dissected, freeing the entire bowel. The hernia content was therefore reduced in the abdomen. The fascia was carefully explored to identify other possible defects. If defects were found, fascial bridges were cut to create a single defect. The fascial defect was measured (length × width). A composite mesh was placed. The dimensions of the mesh must guarantee a 5 cm overlap around the defect. The mesh was placed inside the abdominal cavity with the coated surface facing the bowel. The naked surface faced the abdominal wall fascia and was fixed to it with nonresorbable stitches. The stitches were placed in the four cardinal directions and all around the circumference. The fascia was closed with interrupted resorbable sutures. Haemostasis was checked. A closed suction drain was placed upon the mesh under the fascia. Subcutaneous tissues were closed with 3-0 Vicryl, and the skin was closed with prolene stitches.

### Respiratory measurements

Forced vital capacity (FVC) represents the volume of air that can be forcibly and maximally exhaled out of the lungs after deep inhalation until no more can be expired (usually expressed in litres) [4].

The forced expiratory volume in 1 s (FEV_1_) represents the maximum amount of air that can be exhaled in one second. FEV_1_ reflects limitations in airflow and differentiates between lower airway obstruction and lung volume restriction, decreasing less than FVC in patients with restrictive syndrome [4, 5].

The ratio of FEV1 to FVC indicates the percentage of the total FVC expelled from the lungs during the first second of forced exhalation. The ratio of FEV1 to FVC is a standardised measure for the detection of airflow limitations with high interpatient consistency [4, 5].

The peak expiratory flow (PEF) represents the maximal flow that can be exhaled when blowing out at a steady rate. PEF reflects the strength of the expiratory muscles. The percentage of predicted PEF (%PEF) represents the percentage of predicted normal values after adjustment for the effects of age, weight change and smoking status according to the US National Health and Nutrition Examination Survey [4–6].

## Results

Between January 2015 and December 2016, a total of 180 patients were admitted to the Policlinico “Paolo Giaccone” Hospital and underwent a surgical operation for incisional ventral hernia repair. Sixty-three patients met the inclusion criteria and were considered for inclusion in the study group. Twelve patients did not comply with the follow-up time and were lost to follow-up. Fifty-one patients were finally considered for the study. The mean follow-up time was 48 months (36–60). The mean patient age was 63.3 y.o. (± 14.32), and 62.8% were males. The median BMI was 27 (± 3.5), and 58.8% of patients were current smokers. The most frequent comorbidities were hypertension (66.6%), diabetes (49%), cardiovascular disease (33.3%) and controlled liver disease (11.7%). Patients were classified as ASA II (25.4%), ASA III (70.6%) and ASA IV (4%). The mean operation time was 90.2 (± 45) min, the mean hernia size was 138.8 (± 2.60) cm^2^, and the mean length of the defect was 10 (± 5) cm. The mean in-hospital stay was 6.0 (± 4.0) days. The early wound complication rate was 9.8%; 6% experienced wound infection that was well controlled with antibiotic therapy, 1.9% experienced seroma and 1.9% experienced haematoma, both of which were treated conservatively. The recurrence rate was 4%. The recurrences were assessed by physical examination first and then CT scan. Recurrences were diagnosed at least 1 year after the surgical operation (see Table 1).

**Table 1:** Demographic, intraoperative and postoperative data

Demographic data	
Age (years) (mean)	63.3 (± 14.3)
Sex	
M	62.8%
BMI (kg/m^2^) (median)	27 (± 3.5)
Smokers	58.8%
Comorbidities	
Hypertension (%)	66.6
DM Type 2 (%)	49
Cardiovascular disease (%)	33.3
Controlled liver disease (%)	11.7
ASA score (%)	
II	25.4
III	70.6
IV	4
Intraoperative data	
Operation time (min) (mean)	90.2 (± 45)
Hernia size (cm^2^) (mean)	138.8 (± 2.6)
Length of the defect (mean)	10 (± 5)
Postoperative data	
In-hospital stay (days) (mean)	6 (± 4)
Early wound complication rate	9.8%
Wound infection	6%
Seroma	1.9%
Haematoma	1.9%
Recurrence	4%
Clavien–Dindo	
I	8.6%
II	3.8%

The results of the respiratory outcome after abdominal wall incisional hernia repair are reported in Table 2 and are detailed below.

**Table 2:** Results of the respiratory outcome after abdominal wall hernia repair

	*t*_0_	*t*_1_	*t*_2_	*p*
FVC (mean)	3.17 (1.76–4.13)	3.12 (1.32–3.99)	3.15 (1.35–3.89)	
				0.24
				0.26
FEV1 (mean)	2.39 (1.70–3.06)	2.16 (1.63–3.06)	2.23 (1.67–3.05)	
				0.18
				0.21
PEF (mean)	4.32 (4.03–7.92)	6.7 (4.27–8.24)	6.5 (4.25–8.21)	
				0.012
				0.026
FEV1/FVC (mean)	74 (70–76)	75 (72–78)	75 (72–78)	
				0.41
				0.41
%PEF (mean)	75 (61–89)	87 (76–98)	85 (74–96)	
				0.009
				0.03

The comparison of the mean preoperative (*t*_0_) and postoperative (*t*_1_ and *t*_2_) FVC was not significant [*t*_0_ 3.17 (1.76–4.13), *t*_1_ 3.12 (1.32–3.99) with *p* = 0.24 and *t*_2_ 3.15 (1.35–3.89) with *p* = 0.26], the difference between the preoperative and the postoperative FEV1 [*t*_0_ 2.39 (1.70–3.06), *t*_1_ 2.16 (1.63–3.06) with *p* = 0.18 and *t*_2_ 2.23 (1.67–3.05) with *p* = 0.21] and the difference between the preoperative and postoperative FEV1/FVC ratio [*t*_0_ 74 (70–76), *t*_1_ 75 (72–78) with *p* = 0.41 and *t*_2_ 75 (72–78) with *p* = 0.41] were also not significant.

The difference between preoperative and postoperative PEF was significant [*t*_0_ 4.32 (4.03–7.92), t_1_ 6.7 (4.27–8.24) with *p* = 0.012 and *t*_2_ 6.5 (4.25–8.21) with *p* = 0.026]. The %PEF increased from 75% (61–89%) preoperatively to 87% (76–98%) and 85% (74–96%) postoperatively at the t_1_ and t_2_ points, respectively (*p* = 0.009 and *p* = 0.03, respectively).

## Discussion

The data analysed revealed a significant improvement in expiratory lung function after abdominal wall incisional hernia repair, as reflected by the comparison of the mean preoperative and postoperative PEF and %PEF collected 1 week before the surgical operation (*t*_0_) and 12 months (*t*_1_) and 3 years (*t*_2_) later.

The high incidence rate of abdominal wall incisional hernia after surgical operations justifies the high attention given to this topic by the main surgical societies [1, 5, 7, 8].

All efforts are oriented to better understand the pathogenic mechanisms that cause incisional hernia to prevent it when possible and to perform the best therapy when it occurs, with the aim of offering the best postoperative quality of life (QoL) [8].

Traditionally, the most important outcome after hernia repair has been recurrence of the hernia, followed by postoperative complications and readmissions, and last, the patient-reported outcomes [9]. However, patient-reported outcomes after abdominal wall reconstruction should include not only QoL, pain and other related parameters, but also considerations about postoperative respiratory function recovery [5, 8].

This study represents part of a project that assesses physical function after abdominal wall incisional hernia repair. The review of the literature shows some recent progress in the comprehension of the interactions between abdominal wall surgery and respiratory function [5, 8, 10], but no author has performed a study with long-term follow-up as we have done here.

The measurement timepoints were chosen to avoid bias as much as possible: *t*_0_ was near enough to the surgical operation to observe the most realistic respiratory function and the alterations caused by the abdominal hernia at the time when the patient was ready for the operation. The postoperative *t*_1_ data collection timepoint was 12 months after surgery due to the complete recovery in abdominal wall elasticity and compliance [5, 8]. Finally, the reason for the 3-year follow-up is to observe the respiratory function trend a long time after the surgical operation.

The improvement in respiratory lung function after abdominal wall reconstruction should also be related to the specific surgical technique used. The guidelines are still unclear and not specific about this, so the inhomogeneity of the data is due to the variability in study designs [5, 8]. However, abdominal wall surgery entirely reflects the idea of a tailored surgery, so different approaches should be designed by different surgeons for the same patient and by the same surgeon for different patients. To reduce the related bias, we opted to select one kind of operation performed by a single skilled general surgeon. Therefore, we excluded patients who received tailoured component separation.

Since 2000, the IPOM open technique has been the procedure of choice for ventral hernia repair using a highly standardised technique in highly complex abdominal wall hernias.

The restoration of the abdominal wall anatomy through midline reconstruction and its reinforcement with prosthetic material should be responsible for the observed restoration of respiratory function. However, the relevant influence of the abdominal wall muscles on respiratory dynamics is well known to optimise diaphragm function [7, 11–13]. Their fixation to the ribcage permits a downward pull, assisting forced expiration. As a result, the improvement in respiratory function after abdominal wall reconstruction reflects the spirometric parameter normalisation trend in the reference population [5, 9]. This evidence underlines the importance of linea alba reconstruction in abdominal surgery even though additional studies would be helpful if designed ad hoc comparing functional outcomes between different surgical techniques.

The high incidence of smokers is likely related to the socioeconomic status of the population involved. It has been proven that a higher smoking rate is seen among low socioeconomic status groups [11]. It is also common that as socioeconomic status decreases, the clinical presentation worsens. Adopting a syllogism, the larger incisional ventral hernia is often related to the lower socioeconomic status groups and to the higher incidence of smoking.

According to Jensen et al. [5], we found no significant alterations in pre- and postoperative FVC, FEV_1_ or the FVC/FEV_1_ ratio. This is because these measures reflect the pulmonary volumes that remain unchanged if pulmonary diseases do not occur, and they are independent of the possible modifications of respiratory muscular function and of the force used to generate flow.

In the past, Blatnik et al. [10] demonstrated the correlation between abdominal wall hernia repair and increased plateau pressure during positive-pressure mechanical ventilation (low airway compliance) in patients with large hernia and loss of domain in the immediate postoperative period. However, the absence of variation in FVC and FEV_1_ after the long postoperative follow-up demonstrates the high plasticity of the respiratory tract. This evidence is also confirmed by the results presented by Agnew et al. [14].

Relating respiratory function to incisional hernia repair permitted (1) an understanding of the strong relationship that exists between pulmonary ventilation and the abdominal wall. The assessment of this relationship also helps to guide the correct management of a complex disease that is often underestimated. In fact, the results of the study (2) provide evidence that should help determine surgical indications, guiding a decision in complex scenarios where it is difficult to completely define the risk–benefit balance in critical patients. Moreover, (3) the results should be used to propose a prognostic scoring system to predict the results of the surgical operation in selected patients with risk factors, such as pulmonary comorbidities or high perioperative risk related to respiratory function. This study provides evidence of the existence of a direct correlation between abdominal wall restoration and pulmonary function improvement, although there is a limitation that must be accentuated: the sample size is small, increasing the risk of bias. The results of this study, together with those of other study groups, such as Mommers et al. [15], provide an overview of the relationship between respiratory function and incisional ventral hernia repair. The study is novel since no previous papers have described the long-term effect of abdominal wall reconstruction on respiratory function. The results could impact the daily practice of specialists in incisional hernia repair.

## Conclusions

In conclusion, the study demonstrated that (1) abdominal wall incisional hernia is responsible for significant alterations in respiratory function, (2) abdominal wall incisional hernia repair with linea alba restoration results in a significant improvement in respiratory outcome, restoring the normal anatomy of the abdominal wall, and (3) the improvement gained after the operation is; therefore, maintained over time as demonstrated by the long-term follow-up conducted.

The proposed data provide results to demonstrate the utility of the surgical operation in improving respiratory function in patients with abdominal wall incisional hernia. The study proves the role of abdominal wall anatomy restoration in recovering respiratory functional activity.

This is the first study to assess the trend in respiratory function after a long-term follow-up, demonstrating the usefulness and duration of the effects of abdominal wall reconstruction.

## Data Availability

Not applicable.
